# Supplementary material for: Universal efficiency boost in prethermal quantum heat engines at negative temperature
Source: Nat Commun. 2025 Nov 25;16:10593. doi: 10.1038/s41467-025-66424-1 (PMC12658249; doi:10.1038/s41467-025-66424-1)
Supplement: Supplementary file 1 — Supplementary Information [file 41467_2025_66424_MOESM1_ESM.pdf]

Supplementary Information: Universal efficiency boost in prethermal quantum heat engines at negative temperature

Alberto Brollo, Adolfo del Campo, Alvise Bastianello

The Supplementary Information gathers the more technical, albeit standard, aspects of our article. In particular:

- Supplementary Discussion 1 gives a pedagogical overview of the Ising chain in the transverse field and provides further details on the infinitesimal and finite cycles presented in the main text.
- Supplementary Discussion 2 reviews the thermodynamic Bethe ansatz approach to the XXZ model and further analyzes infinitesimal and finite cycles.
- Supplementary Methods 1 discusses the numerical schemes used to solve the thermodynamics and hydrodynamics of integrable models.

SUPPLEMENTARY DISCUSSION 1. THE QUANTUM ISING CHAIN IN TRANSVERSE FIELD

The one-dimensional transverse Ising model can be mapped onto a system of free spinless fermions via a Jordan-Wigner transformation. See Supplementary Ref.[1] for a pedagogical review. In this section, we outline this mapping and demonstrate how the resulting simplified thermodynamic description allows for solving both thermal and prethermal cycles in both the infinitesimal and finite cases. In particular, we derive the full analytical solution for the infinitesimal cycle, providing a concrete example of the general formulas in Eqs. (5) and (6).

Jordan-Wigner mapping to free fermions — The Hamiltonian of the transverse one-dimensional Ising model (7) is given by

$$H_{\text{Ising}} = -\sum_j \left(\sigma^x_{j+1}\sigma^x_j + h\sigma^z_j\right)\,, \qquad \text{(Supplementary Equation 1)}$$

where $\sigma^{x,y,z}$ are the standard Pauli matrices. The single-site Hilbert space is two-dimensional, and the two states are connected by the $SU(2)$ ladder operators $\sigma^\pm = (\sigma^x \pm i\sigma^y)/2$. One can introduce fermionic creation/annihilation operators, $c_i^\dagger, c_i$, via the Jordan-Wigner transformation [1] $\exp\{i\pi\sum_{i<j} c_i{}^\dagger c_i\} c_j{}^\dagger = \sigma_j^+$, which satisfy the canonical anticommutation relations, $\{c_j^\dagger, c_i\} = \delta_{i,j}$ and $\{c_j^\dagger, c_i^\dagger\} = \{c_j, c_i\} = 0$. This transformation maps the Hamiltonian into

$$H = -\sum_j \left(c_j^\dagger c_{j+1} + c_j^\dagger c_{j+1}^\dagger + \text{h.c.}\right) + h\sum_{j=-L}^{L}\left(2c_j^\dagger c_j - 1\right). \qquad \text{(Supplementary Equation 2)}$$

This Hamiltonian is quadratic and can be diagonalized in the Fourier basis through a Bogoliubov rotation:

$$\begin{pmatrix} c_j \\ c_j^\dagger \end{pmatrix} = \int_{-\pi}^{\pi} \frac{\mathrm{d}\lambda}{\sqrt{2\pi}} e^{i\lambda j} U_{\theta_\lambda} \begin{pmatrix} \gamma(\lambda) \\ \gamma^\dagger(-\lambda) \end{pmatrix}, \qquad U_{\theta_\lambda} = \begin{pmatrix} \cos\theta_\lambda & i\sin\theta_\lambda \\ i\sin\theta_\lambda & \cos\theta_\lambda \end{pmatrix} \qquad \text{(Supplementary Equation 3)}$$

where $\gamma(\lambda)$ are canonical fermionic operators $\{\gamma(\lambda), \gamma^\dagger(\mu)\} = \delta(\lambda - \mu)$. The angle θ_λ parameterizes the Bogoliubov rotation, and the choice $\theta_\lambda = -\frac{1}{2i}\log\left(\frac{h - e^{i\lambda}}{(\cos\lambda - h)^2 + \sin^2\lambda}\right)$ diagonalizes the Hamiltonian as $H(h) = \int \mathrm{d}\lambda\, e(\lambda, h)\gamma^\dagger(\lambda)\gamma(\lambda) + \text{const.}$ with $e(\lambda, h) = 2\sqrt{(\cos\lambda - h)^2 + \sin^2\lambda}$ within the Brillouin zone $[-\pi, \pi]$. In contrast with the main text, we make the dependence on h explicity for the sake of clarity. From the new fermionic ladder operators, an infinite number of conserved quantities can be constructed $n(\lambda) = \gamma^\dagger(\lambda)\gamma(\lambda)$ [1].

GGEs and adiabatic flow equations — A thermal state at inverse temperature β is described by the Fermi-Dirac momentum density $\rho(\lambda, h) = \frac{1}{2\pi}\left(1 + e^{\beta e(\lambda,h)}\right)^{-1}$. It can be shown that any momentum density $\rho(\lambda, h)$ uniquely corresponds to a generalized Gibbs ensemble (GGE) [1]. In particular, if $\rho(\lambda, h)$ cannot be expressed in the Fermi-Dirac functional form, it corresponds to a non-thermal GGE. According to Eq. (2), the evolution of β along the thermal adiabatic stroke is given by $\langle H^2\rangle_c \partial_h\beta + \langle H\partial_h H\rangle_c \beta = 0$, where the correlators are computed as in Eq.

(12). The prethermal adiabatic flow is instead governed by the unitary evolution of the system. In fact, because of the diagonalization of the Hamiltonian, the chain behaves as a system of decoupled harmonic oscillators to which the adiabatic theorem applies. Consequently, the momentum density remains invariant along the prethermal flow. In particular, the application of the generalized hydrodynamics (GHD) equation (9) to the Ising chain also gives stationarity since the effective force vanishes, in agreement with the adiabatic theorem.

Infinitesimal cycles — In order to compare the cycles, we compute the energy difference between thermal and prethermal strokes starting from a thermal state at external magnetic field h_0 and inverse temperature $\beta(h_0)$. The internal energy during the stroke along the prethermal states is given by $\langle H(h)\rangle^{\rm pth} = \int_{-\pi}^{\pi} {\rm d}\lambda\, e(\lambda,h)\frac{1}{2\pi}\left(1+e^{\beta(h_0)e(\lambda,h_0)}\right)^{-1}$, whereas for the thermal case, $\langle H(h)\rangle^{\rm th} = \int_{-\pi}^{\pi}{\rm d}\lambda\, e(\lambda,h)\rho(\lambda,h)$ where $\rho(\lambda,h) = \frac{1}{2\pi}\left(1+e^{\beta(h)e(\lambda,h)}\right)^{-1}$ and $\beta(h)$ is the solution of the thermal adiabatic flow equation. When expanding in small strokes of size δh, the difference at first order is zero. We obtain the second-order correction

$$\langle H(h_0+\delta h)\rangle^{\rm pth} - \langle H(h_0+\delta h)\rangle^{\rm th} = -\frac{\delta h^2}{2}\int_{-\pi}^{\pi}\frac{{\rm d}\lambda}{2\pi}\left(2\partial_h e(\lambda,h_0)\partial_h\rho(\lambda,h_0) + e(\lambda,h_0)\partial_h^2\rho(\lambda,h_0)\right). \quad \text{(Supplementary Equation 4)}$$

One then integrates by parts the second term and uses explicitly the flow equation to evaluate the first derivative of the momentum density. Recollecting everything in terms of correlators (see Sec.).

$$\langle H(h_0+\delta h)\rangle^{\rm pth} - \langle H(h_0+\delta h)\rangle^{\rm th} = \beta\frac{\delta h^2}{2}\langle\partial_h H|\mathbb{P}^{\rm pth}|\partial_h H\rangle\left(1-\frac{|\langle H\partial_h H\rangle_{\rm c}|^2}{\langle H^2\rangle_{\rm c}\langle\partial_h H|\mathbb{P}^{\rm pth}|\partial_h H\rangle}\right), \quad \text{(Supplementary Equation 5)}$$

where the correlators are clearly computed in the initial thermal state and $\langle\partial_h H|\mathbb{P}^{\rm pth}|\partial_h H\rangle = \int_{-\pi}^{\pi}{\rm d}\lambda\,(\partial_h e(\lambda,h_0))^2\frac{1}{2\pi}\left(1+e^{\beta(h_0)e(\lambda,h_0)}\right)^{-1}$ (see Supplementary Equation 20).

To compare this formula with Eq. (6), which expresses the difference between the extracted works, we must also consider the stroke that starts from the other reservoir in the infinitesimal cycle, at $(h_0+\delta h, \beta(h_0)+\delta\beta)$. The expression for the energy difference at the end of this stroke remains the same, except that the correlators must be evaluated at $(h_0+\delta h,\beta(h_0)+\delta\beta)$, and the stroke direction is reversed, $\delta h\to-\delta h$. The first modification introduces only subleading corrections, while the second leaves the formula unchanged. Taking everything into account, the work difference $\delta\mathcal{W} = \mathcal{W}^{\rm pth} - \mathcal{W}^{\rm th}$ is simply minus twice the previous expression, in full agreement with Eq. (6). In this case, where the thermal space is one-dimensional, it appears immediately that the sign of the above formula depends exclusively on the temperature. The autocorrelator and $\langle\partial_h H|\mathbb{P}^{\rm pth}|\partial_h H\rangle$ are clearly positive, and the term in round brackets can be bounded using a simple Cauchy-Schwarz inequality. With similar computations, one can derive the leading contributions to the extracted work either from the thermal or prethermal cycle

$$\mathcal{W}^{\rm th} = -\delta h\delta\beta\langle H\partial_h H\rangle_{\rm c} - \beta\delta h^2\frac{|\langle H\partial_h H\rangle_{\rm c}|^2}{\langle H^2\rangle_{\rm c}}, \qquad \mathcal{W}^{\rm pth} = -\delta h\delta\beta\langle H\partial_h H\rangle_{\rm c} - \beta\delta h^2\langle\partial_h H|\mathbb{P}^{\rm pth}|\partial_h H\rangle. \quad \text{(Supplementary Equation 6)}$$

Notice that the second term of the thermal work can be written using the thermal projector, which is one-dimensional in this case: $\mathbb{P}^{\rm th} = [\langle H^2\rangle_{\rm c}]^{-1}|H\rangle\langle H|$. Specifically, it takes the form $\langle\partial_h H|\mathbb{P}^{\rm th}|\partial_h H\rangle$ (see Methods in the main text). In general, the work depends on the shape of the cycle and cannot be strictly bounded to be larger than zero, as required for a heat engine. A simplification is introduced if we consider skewed cycles where $|\delta\beta|\gg|\delta h|$. Consequently, the difference between the two cycles becomes subleading; the prethermal work coincides with the thermal one $\mathcal{W}^{\rm pth}\simeq\mathcal{W}^{\rm th}\simeq-\delta h\delta\beta\langle H\partial_h H\rangle_{\rm c}$, and this can always be made positive by tuning the sign of the variations. Furthermore, the heat absorbed in a skewed cycle is given by $\mathcal{Q}_{\rm abs}\simeq|\delta\beta|\langle H^2\rangle_{\rm c}$, and at leading order does not distinguish between the two cycles. The heat engine efficiency in the skewed thermal cycle takes a particularly simple form

$$\eta^{\rm th}\simeq\frac{|\delta h\langle H\partial_h H\rangle_{\rm c}|}{\langle H^2\rangle_{\rm c}}, \quad \text{(Supplementary Equation 7)}$$

and the prethermal efficiency differs from the thermal one according to

$$\frac{\eta^{\rm pth}}{\eta^{\rm th}}\simeq 1-\left|\frac{\delta h}{\delta\beta}\right|\beta\frac{\langle\partial_h H|\mathbb{P}^{\rm pth}|\partial_h H\rangle}{|\langle H\partial_h H\rangle_{\rm c}|}\left(1-\frac{|\langle H\partial_h H\rangle_{\rm c}|^2}{\langle H^2\rangle_{\rm c}\langle\partial_h H|\mathbb{P}^{\rm pth}|\partial_h H\rangle}\right). \quad \text{(Supplementary Equation 8)}$$

The last two formulas are plotted in panels a) and b) of Fig. 2 in the main text. In these plots, only negative temperature is reported, since due to particle-hole symmetry of free fermionic theories on the lattice, at positive temperature, the plot would be the same, with a reversed sign in the one for the relative efficiency.

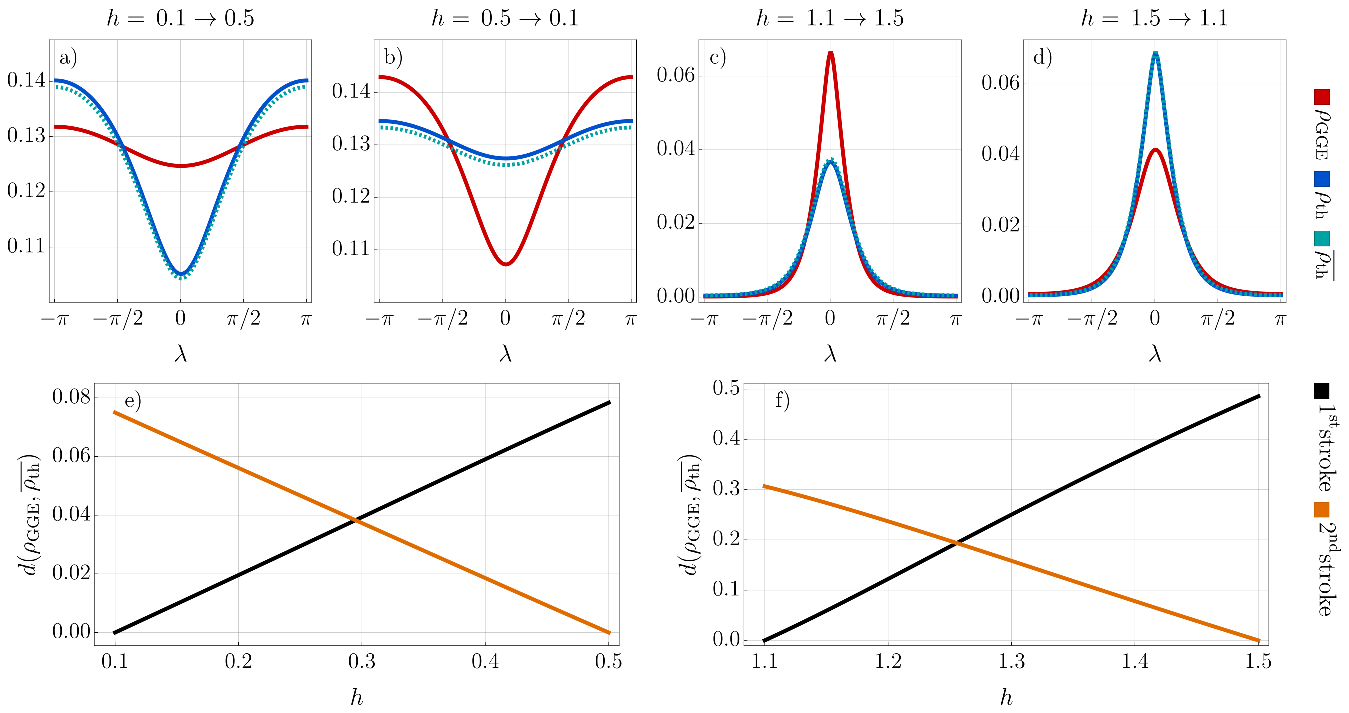

Supplementary Fig. 1. **Comparison between the momentum distributions in the Ising model at the end of finite strokes and relative distance of GGEs from equilibrium.** Panel (a-d): Ising model's momentum distributions $\rho(\lambda)$ at the end of the strokes in the cycles in Fig. 3 of the main text. In each panel, three momentum distributions are plotted: ρ_{GGE} is the one unitary evolved, equal to the initial thermal state due to the adiabatic theorem. The final state along the thermal stroke is ρ_{th}, while $\overline{\rho_{\mathrm{th}}}$ is thermal with the same energy as the prethermal one. The latter is useful both for quantifying non-ergodicity and comparing the two cycles. Panels a) and b) refer to strokes in the negative temperature cycle, as one can see since the more energetic modes are excited, while panels c) and d) refer to the positive temperature cycle. In this case, despite the thermal momentum distributions are not very different, an appreciable energy difference appears (see Fig. 3. Panel (e,f): evolution along the stroke of the GGE's distance from equilibrium, measured as the relative distance in the L_2 norm between ρ_{GGE} and $\bar{\rho}_{\mathrm{th}}$. For the explicit formula, see Supplementary Equation 9. The orange and the black lines refer to the two different strokes of the prethermal cycle. The maximum relative distance goes from the 8% of the strokes at negative temperature, up to 50% in the first stroke at positive temperature, meaning the system is considerably driven out of equilibrium.

Finite cycles — Finite cycles are numerically solved according to Section . In the case of the Ising chain, the solution of the prethermal cycle is particularly simple since the momentum density does not evolve. The results are reported in Fig. 3 of the main text. The results for infinitesimal cycles guided us in identifying the regions of the parameter space where the difference between the two strokes was greater. In particular, we find as the most informative quantity $\delta\mathcal{W}/(\delta h\langle\partial_h H\rangle)$, which tells how much the thermal and prethermal strokes differ from each other, relative to the energy change. Based on this reasoning, we identify the cycles reported in the main text.

As a complement to those plots, Supplementary Fig. 1 presents a comparison of the system's momentum distributions at the end of each stroke. For the two strokes at negative temperature, the difference is more pronounced, as expected from the energy difference shown in Fig. 3. For the strokes at positive temperature, we selected a set of parameters that produces a milder difference. A larger discrepancy would have resulted in negative extracted work in the prethermal cycle, meaning that it would no longer work as a heat engine. Even with these small differences, the efficiency of the thermal cycle remains an order of magnitude higher. Furthermore, we present in Supplementary Fig. 1 the evolution along the prethermal strokes of the GGE's distance from equilibrium, measured as the relative distance in the L_2 norm between $\rho(\lambda)$ describing the GGE and $\bar{\rho}_{\mathrm{th}}(\lambda)$ of a thermal state with the same energy than the GGE (i.e. the thermal state one would relax to from the GGE without further work injection). Explicitly, the formula takes the form

$$d(\rho_{\mathrm{GGE}},\bar{\rho}_{\mathrm{th}}) = \left(\frac{\int \mathrm{d}\lambda[\rho_{\mathrm{GGE}}(\lambda)-\bar{\rho}_{\mathrm{th}}(\lambda)]^2}{\int \mathrm{d}\lambda[\bar{\rho}_{\mathrm{th}}(\lambda)]^2}\right)^{1/2}. \qquad \text{(Supplementary Equation 9)}$$

From these plots we can read that the system is considerably driven out of equilibrium: at the end of the strokes at

negative temperature the relative distance is around the 8%, and for the first stroke at positive temperature goes up to 50%. These plots give a quantitative evidence that we are considering a genuine nonequilbrium phenomenon.

We conclude this section by noting that although the bound on infinitesimal cycles does not constitute formal proof, we have tested a large number of finite cycles and consistently found that prethermal cycles are more efficient at negative temperatures and vice versa at positive temperatures.

SUPPLEMENTARY DISCUSSION 2. THE XXZ SPIN CHAIN IN EASY-AXIS REGIME

In this section, we provide an overview of the thermodynamics and GHD of the XXZ spin chain, discuss the equations for infinitesimal cycles, and further elaborate on finite cycles.

Thermodynamic Bethe ansatz — We refer mainly to Supplementary Ref. [2]; see also Supplementary Ref. [3] for conventions. The Hamiltonian is given by

$$H_{\mathrm{XXZ}} = -J\sum_j \left(\sigma^x_{j+1}\sigma^x_j + \sigma^y_{j+1}\sigma^y_j + \Delta\sigma^z_{j+1}\sigma^z_j\right), \qquad \text{(Supplementary Equation 10)}$$

where $\sigma^{x,y,z}$ are the standard Pauli matrices. For anisotropy values $|\Delta| > 1$, spin alignment is favored along the z-axis in the so-called easy-axis regime. The sign of $J\Delta$ distinguishes between the ferromagnetic ground state (if positive) and the Néel state (if negative). The many-body Hilbert space can be described in terms of excitations over the ferromagnetic ground state. The fundamental excitations are magnons –isolated and delocalized spin flips– and bound states thereof, which within the TBA framework are referred to as “Bethe strings” or “strings”, in short. From the perspective of the Thermodynamic Bethe Ansatz (TBA) [2], each string is treated as a distinct quasiparticle species, each associated with its own root density $\{\rho_j(\lambda)\}_{j=1}^{\infty}$, filling function $\{\vartheta_j(\lambda)\}_{j=1}^{\infty}$, and pseudoenergy $\{\varepsilon_j(\lambda)\}_{j=1}^{\infty}$. The system is thus described by coupled TBA equations, where the scattering phase is generalized to a matrix $\Theta_{j,k}(\lambda)$ that accounts for the scattering between different types of strings.

We focus on the case with $J = -1$ and $\Delta > 1$, as the Hamiltonian is symmetric under $(J,\Delta) \to (-J,-\Delta)$, meaning that for other parameter choices, the eigenproblem remains the same except for a possible sign change in energy eigenvalues. The expectation value of the conserved charges is computed by summing the contributions of each string. As an example, the energy and momentum densities for each string species are given by

$$e_j(\lambda) = -\frac{1}{2}\sinh(\theta)\partial_\lambda p_j(\lambda), \qquad p_j(\lambda) = 2\arctan\left[\coth\left(\frac{j\theta}{2}\right)\tan\lambda\right], \qquad \text{(Supplementary Equation 11)}$$

where the rapidities are constrained within the Brillouin zone $\lambda \in [-\pi/2, \pi/2]$, and the angle θ parameterizes the anisotropy as $\Delta = \cosh\theta$. The expectation values of the Hamiltonian and local magnetization are then expressed as

$$\frac{1}{L}\langle H\rangle = \frac{J\Delta}{4} + \sum_j \int_{-\pi/2}^{\pi/2} \mathrm{d}\lambda\, e_j(\lambda)\rho_j(\lambda), \qquad \langle\sigma^z_i\rangle = 1 - \sum_j \int_{-\pi/2}^{\pi/2} \mathrm{d}\lambda\, j\rho_j(\lambda), \qquad \text{(Supplementary Equation 12)}$$

where L is the length of the chain and $N = L - \sum_i \sigma^z_i$ is the magnons' number operator, which commutes with the Hamiltonian. Above, $J\Delta/4$ is the energy of the fully polarized state: since a state-independent offset does not affect thermodynamics, neither work nor exchanged heat, we herein neglect this additional term. In general, higher-order correlators require summing the contributions from all string species. The explicit form of the scattering phase is

$$\Theta_{j,k}(\lambda) = (1-\delta_{j,k})\frac{p_{|j-k|}(\lambda)}{2\pi} + \frac{p_{j+k}(\lambda)}{2\pi} + 2\sum_{\ell=1}^{\min(j,k)-1}\frac{p_{|j-k|+2\ell}(\lambda)}{2\pi}. \qquad \text{(Supplementary Equation 13)}$$

Adiabatic flow equations — In this para, we derive the expressions for the correlators required to compute the exact flow equations (2). To simplify the notation, we consider formulas for a generic integrable system without Bethe strings: the final results are easily generalized to the XXZ model by adding a summation over the quasiparticle species. Starting from the expectation value of a conserved charge $\frac{1}{L}\langle Q_j\rangle = \int \mathrm{d}\lambda q_j(\lambda)\rho(\lambda)$, this can be rewritten as

$$\frac{1}{L}\langle Q_j\rangle = \int \mathrm{d}\lambda\mathrm{d}\lambda' q_j(\lambda)[\vartheta^{-1} + \tfrac{1}{2\pi}\varphi]^{-1}_{(\lambda,\lambda')}\frac{1}{2\pi}\partial_{\lambda'}p \qquad \text{(Supplementary Equation 14)}$$

since $\rho(\lambda) = \vartheta(\lambda)\frac{(\partial_\lambda p)^{\rm dr}}{2\pi}$ and $[1+\frac{1}{2\pi}\varphi\vartheta]^{-1}_{(\lambda,\lambda')}$ is the dressing integral kernel operator. The connected two-point correlator of two charges is obtained as $\langle Q_i Q_j\rangle_{\rm c} = -\partial_{\beta_i}\langle Q_j\rangle$. The derivation acts only on the integral kernel as

$$\frac{1}{L}\partial_{\beta_i}\langle Q_j\rangle = \int {\rm d}\lambda {\rm d}\lambda' {\rm d}\lambda'' q_j(\lambda)[\vartheta^{-1}+\tfrac{1}{2\pi}\varphi]^{-1}_{(\lambda,\lambda'')}\frac{\partial_{\beta_i}\vartheta(\lambda'')}{\vartheta^2(\lambda'')}[\vartheta^{-1}+\tfrac{1}{2\pi}\varphi]^{-1}_{(\lambda'',\lambda')}\frac{1}{2\pi}\partial_{\lambda'}p\,. \quad \text{(Supplementary Equation 15)}$$

The derivative of the filling is $\partial_{\beta_i}\vartheta = -\vartheta(1-\vartheta)q_i^{\rm dr}$, which can be verified by differentiating the TBA equation. The two integral kernels, combined with $\vartheta^{-2}(\lambda'')$, act as dressing operations, respectively, on the left and on the right. Summing everything, we obtain

$$\tfrac{1}{L}\langle Q_i Q_j\rangle_{\rm c} \overset{L\to\infty}{\simeq} \int {\rm d}\lambda\, q_i^{\rm dr}\rho(1-\vartheta)q_j^{\rm dr}, \quad \text{(Supplementary Equation 16)}$$

where $\simeq$ denotes equality in the thermodynamic limit. The derivation of the susceptibility matrix follows similar steps. The expectation value of the derivative of a charge is $\frac{1}{L}\langle\partial_\chi Q_j\rangle \simeq \int {\rm d}\lambda\left(\partial_\chi q_j\rho + \frac{1}{2\pi}\partial_\lambda q_j f^{\rm dr}\vartheta\right)$ [3] , and similarly to before, can be rewritten as

$$\tfrac{1}{L}\langle\partial_\chi Q_j\rangle \simeq \int {\rm d}\lambda {\rm d}\lambda'\frac{1}{2\pi}\left(\partial_\chi q_j[\vartheta^{-1}+\tfrac{1}{2\pi}\varphi]^{-1}_{(\lambda,\lambda'')}\partial_{\lambda'}p + \partial_\lambda q_j[\vartheta^{-1}+\tfrac{1}{2\pi}\varphi]^{-1}_{(\lambda,\lambda'')}f\right). \quad \text{(Supplementary Equation 17)}$$

The derivative follows the same steps as before but introduces an extra term because $f(\lambda) = -\partial_\chi p(\lambda) + \int {\rm d}\lambda'\partial_\chi\Theta(\lambda-\lambda')\vartheta(\lambda')(\partial_{\lambda'}p)^{\rm dr}(\lambda')$ depends on the Lagrange multiplier via the filling inside the integral

$$-\tfrac{1}{L}\partial_{\beta_i}\langle\partial_\chi Q_j\rangle \simeq \int {\rm d}\lambda\rho(1-\vartheta)q_i^{\rm dr}\left((\partial_\chi q_j)^{\rm dr} + \frac{(\partial_\lambda q_j)^{\rm dr}}{(\partial_\lambda p)^{\rm dr}}f^{\rm dr}\right) + \int {\rm d}\lambda(\partial_\lambda q_j)^{\rm dr}\partial_{\beta_i}f\vartheta\,. \quad \text{(Supplementary Equation 18)}$$

Explicitly computing $\partial_{\beta_i}f(\lambda)$, the second integral can be absorbed into the first using $\Lambda_j(\lambda) = -\partial_\chi q_j(\lambda) + \int {\rm d}\lambda'\partial_\chi\Theta(\lambda-\lambda')\vartheta(\lambda')(\partial_{\lambda'}q_j)^{\rm dr}(\lambda')$, leading to

$$\tfrac{1}{L}\langle Q_i\partial_\chi Q_j\rangle_{\rm c} \simeq \int {\rm d}\lambda\, q_i^{\rm dr}\rho(1-\vartheta)\left(f^{\rm dr}\frac{(\partial_\lambda q_j)^{\rm dr}}{(\partial_\lambda p)^{\rm dr}} - \Lambda_j^{\rm dr}\right). \quad \text{(Supplementary Equation 19)}$$

Although we can compute the flow equations exactly, evaluating the prethermal work is cumbersome due to the infinite-dimensional space of conserved charges in integrable systems. However, we can use hydrodynamic projections and consider the more accessible formula $\left[A^T_{\rm pth}C^{-1}_{\rm pth}A_{\rm pth}\right]_{i,j} = \langle\partial_\chi Q_i|\mathbb{P}^{\rm pth}|\partial_\chi Q_j\rangle$, where the scalar product is defined in the space of conserved charges as $\langle Q_i|Q_j\rangle \equiv \langle Q_i Q_j\rangle_{\rm c} = L[C_{\rm pth}]_{i,j}$, and the projector is $\mathbb{P}^{\rm pth} = \sum_{i,j}[C^{-1}_{\rm pth}]_{i,j}|Q_i\rangle\langle Q_j|$. It is convenient to move to a rapidity basis $|\lambda\rangle$ with the definition $\langle Q_i|\lambda\rangle = Lq_i(\lambda)$, then in this basis the covariance matrix is written as $\langle\lambda'|C_{\rm pth}|\lambda\rangle = \int {\rm d}\lambda''[1+\frac{1}{2\pi}\vartheta\varphi]^{-1}_{(\lambda',\lambda'')}\rho(\lambda'')(1-\vartheta(\lambda''))[1+\frac{1}{2\pi}\varphi\vartheta]^{-1}_{(\lambda'',\lambda)}$. In this basis, it is easy to invert C and by considering its action on Supplementary Equation 19, it follows that

$$\langle\partial_\chi Q_i|\mathbb{P}^{\rm pth}|\partial_\chi Q_j\rangle = \int {\rm d}\lambda\left(f^{\rm dr}\frac{(\partial_\lambda q_i)^{\rm dr}}{(\partial_\lambda p)^{\rm dr}} - \Lambda_i^{\rm dr}\right)\rho(1-\vartheta)\left(f^{\rm dr}\frac{(\partial_\lambda q_j)^{\rm dr}}{(\partial_\lambda p)^{\rm dr}} - \Lambda_j^{\rm dr}\right). \quad \text{(Supplementary Equation 20)}$$

Infinitesimal cycles — Compared to the Ising case, the equations for the XXZ chain should also take into account the conservation of total magnetization, even for thermal evolution. Before stating the results for this specific case, let us go back to the general case discussed in the main text, where the prethermal cycle conserves N charges, the thermal one $N' < N$, and the tunable parameter is χ. From Eq. (5) follows that the extracted works are

$$\mathcal{W}^{\rm th/pth} = \delta\chi\delta\beta_i\partial_{\beta_i}\langle\partial_\chi H\rangle - \beta(\delta\chi)^2\left[A^T_{\rm th/pth}C^{-1}_{\rm th/pth}A_{\rm th/pth}\right]_{H,H}, \quad \text{(Supplementary Equation 21)}$$

where the notation is the same as in Eq. (6) and we sum over repeated indices up to N', since the system can only exchange the thermal charges with an external reservoir. In the assumption that reservoirs exchange only energy with the system and not other conserved charges, the result is further simplified. If so, the $\delta\beta_i$ are not independent, as we must impose to conserve the thermal charges between the two strokes. Hence, $\delta\beta_i\partial_{\beta_i}\langle Q_j\rangle = 0$ with $j = 2,\ldots,N'$, where the energy $Q_1 \equiv H$ is excluded. The XXZ chain conserves only the number of magnons (i.e., the magnetization) in addition to the energy, obtaining

$$\mathcal{W}^{\rm th/pth} = -\delta\chi\delta\beta\left(\langle H\partial_\Delta H\rangle_{\rm c} - \frac{\langle N\partial_\Delta H\rangle_{\rm c}\langle NH\rangle_{\rm c}}{\langle N^2\rangle_{\rm c}}\right) - \beta(\delta\chi)^2\left[A^T_{\rm th/pth}C^{-1}_{\rm th/pth}A_{\rm th/pth}\right]_{H,H}, \quad \text{(Supplementary Equation 22)}$$

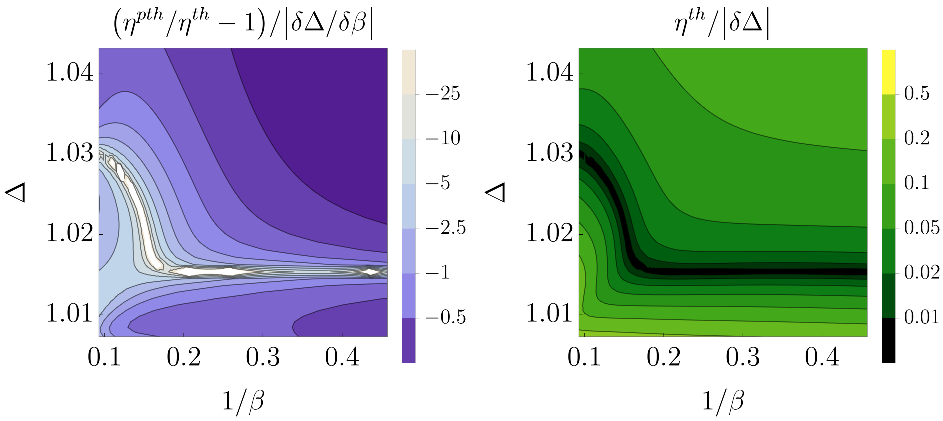

Supplementary Fig. 2. **Thermal vs Prethermal infinitesimal Otto cycle in the XXZ chain.** Relative efficiency of thermal-prethermal infinitesimal skewed Otto cycles for the easy-axis antiferromagnetic $J=-1$ XXZ chain from Supplementary Equation 24. The plot is at a small fixed average magnetization $\langle\sigma^z\rangle = 0.05$ to enhance the string formation at small positive temperatures. As for negative temperatures, regions of large enhancement correspond to vanishing thermal efficiency.

where we identify $\delta\beta \equiv \delta\beta_1$. Notice that this result can be consistently obtained by a brute-force expansion of the GHD and TBA equations, as it should. However, this route is longer than the derivation shown above, and therefore, we do not report it. As discussed in the Methods section of the main text, the second term of the work can be expressed in terms of projectors onto the space of conserved charges, i.e., $\left[A^T_{\rm th/pth}C^{-1}_{\rm th/pth}A_{\rm th/pth}\right]_{H,H} = \langle\partial_\Delta H|\mathbb{P}^{\rm pth/pth}|\partial_\Delta H\rangle$. As we did for the Ising model in Section , we focus on skewed cycles to achieve further simplifications. The leading absorbed heat does not distinguish the two cycles and is given by $\mathcal{Q}_{\rm abs} \simeq |\delta\beta_i\partial_{\beta_i}\langle H\rangle|$, and the infinitesimal efficiencies become

$$\eta^{\rm th} \simeq \frac{\left|\delta\Delta\left(\langle H\partial_\Delta H\rangle_{\rm c} - \frac{\langle N\partial_\Delta H\rangle_{\rm c}\langle NH\rangle_{\rm c}}{\langle N^2\rangle_{\rm c}}\right)\right|}{\langle HH\rangle_{\rm c} - \frac{|\langle NH\rangle_{\rm c}|^2}{\langle N^2\rangle_{\rm c}}}, \qquad \text{(Supplementary Equation 23)}$$

and

$$\frac{\eta^{\rm pth}}{\eta^{\rm th}} \simeq 1 - \left|\frac{\delta\Delta}{\delta\beta}\right|\beta\frac{\langle\partial_\Delta H|\mathbb{P}^{\rm pth}|\partial_\Delta H\rangle}{\left|\langle H\partial_\Delta H\rangle_{\rm c} - \frac{\langle N\partial_\Delta H\rangle_{\rm c}\langle NH\rangle_{\rm c}}{\langle N^2\rangle_{\rm c}}\right|}\left(1 - \frac{|\langle H\partial_\Delta H\rangle_{\rm c}|^2}{\langle H^2\rangle_{\rm c}\langle\partial_\Delta H|\mathbb{P}^{\rm pth}|\partial_\Delta H\rangle} - \frac{|\langle N\partial_\Delta H\rangle_{\rm c}|^2}{\langle N^2\rangle_{\rm c}\langle\partial_\Delta H|\mathbb{P}^{\rm pth}|\partial_\Delta H\rangle}\right),$$

(Supplementary Equation 24)

and again it is easy to see that the prethermal is more efficient at negative temperature due to the Bessel inequality. All terms appearing in the above formula are explicitly reported in Section .

These formulas are those plotted in Fig.2. In addition, Supplementary Fig. 2 shows a plot for positive temperature, illustrating that the thermal cycle is more efficient than the prethermal one. The regions where the differences are most striking correspond to those with vanishing thermal efficiency. Compared to the negative temperature case, we have decreased the average magnetization to $\langle\sigma^z\rangle = 0.05$ to increase the number of magnons and facilitate string formation. Since strings are thermally activated and the system conserves the total number of excitations for each string independently during the dynamics, the presence of more strings drives the system further from a thermal state, thereby enhancing the differences.

Finite cycles — In Fig. 3, for the plot at positive temperature, we have moved into the region of negative anisotropy Δ. Although infinitesimal cycles at positive Δ show a noticeable difference (see Supplementary Fig. 2), we did not find finite cycles in which the prethermal-thermal difference was appreciable. We believe this is because achieving a sufficient number of strings to drive the system significantly away from a thermal state requires increasing the number of magnons, and consequently, the energy. As a result, the relative difference between prethermal and thermal cycles became too small. To circumvent this, we shifted our analysis to the region of negative Δ. Due to the symmetry under $(J,\Delta) \to (-J,-\Delta)$, this choice is equivalent to considering the case $J=+1$ and $\Delta > 1$. Under this transformation, the energy eigenvalues reverse their sign while the eigenvectors remain unchanged. From a thermodynamic perspective, this corresponds to reversing the sign of the temperature. This allowed us to activate more strings while keeping the energy low, even at a positive temperature.

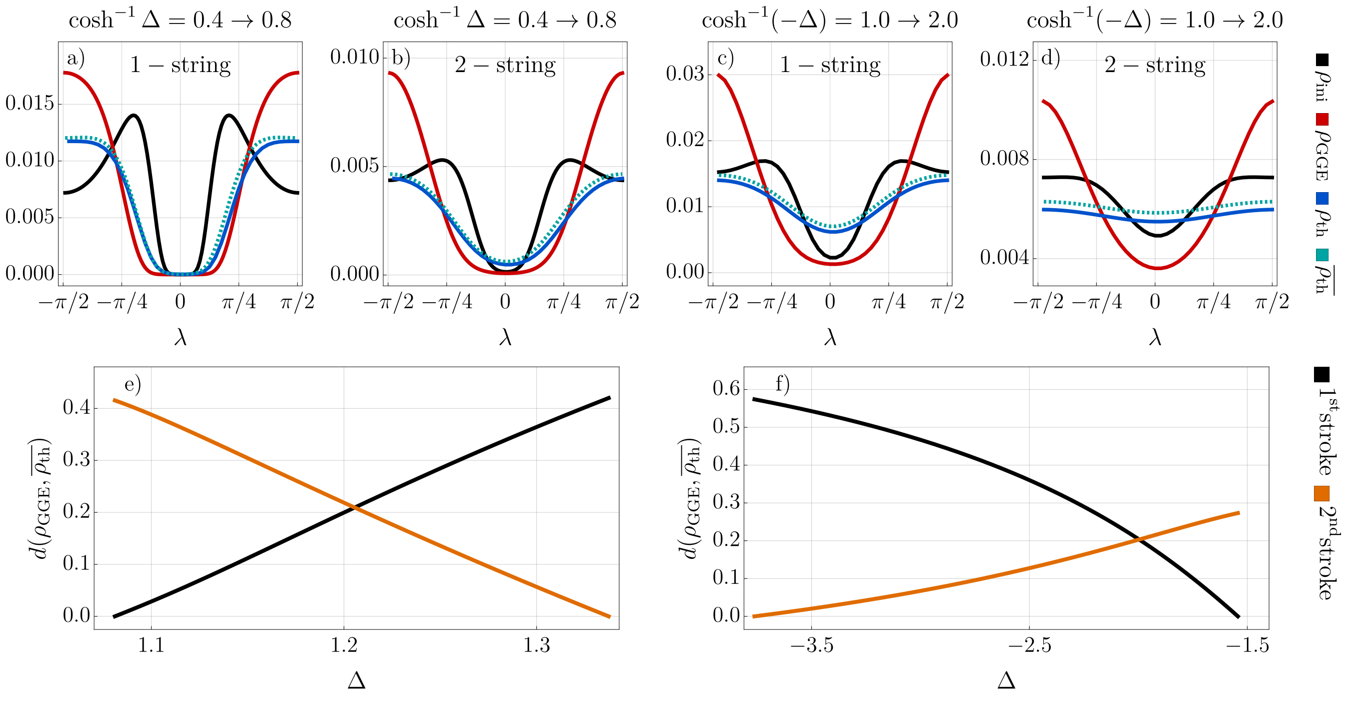

Supplementary Fig. 3. **Comparison between root densities in the XXZ chain at the end of finite strokes and relative distance of GGEs from equlibrium.** . Panels (a) and (b) show the root densities for the first two strings, respectively, in the lowest energy stroke of the negative temperature cycle in Fig. 3. Panels (c) and (d) display the same quantities for the positive temperature cycle. In each panel, four root densities are plotted: $\rho_{\rm ini}$ is the initial thermal state, $\rho_{\rm GGE}$ is the one unitary evolved, and $\rho_{\rm th}$ is the final state along the thermal stroke. The dashed lines represent $\overline{\rho_{\rm th}}$, the root density corresponding to a thermal state with the same energy and magnetization as the one unitarily evolved via GHD. Even from the first two strings, it is evident that bound states are suppressed with their size. Notably, higher modes remain excited even at positive temperature due to the unitary equivalence between $(J = -1, \Delta < -1)$ and $(J = 1, \Delta > 1)$. Panel (e,f): evolution along the stroke of the GGE's distance from equilibrium, measured as the relative distance in the L_2 norm between $\rho_{\rm GGE}$ and $\bar{\rho}_{\rm th}$. The explicit formula is reported in the text. The orange and the black lines refer to the two different strokes of the prethermal cycle. The maximum relative distance goes from the 40% of the strokes at negative temperature, up to 60% in the first stroke at positive temperature, meaning the system is considerably driven out of equilibrium.

As an additional complement, we present in Supplementary Fig. 3 a plot of the evolution of the root densities along the strokes. The figure illustrates that during the adiabatic increase of Δ, excitations shift toward larger rapidities. Moreover, even though the magnetization is fixed across all the states shown in the figure, the area under the root densities following thermal states appears to vary between them. This discrepancy arises because the figure only displays the first two strings, and the contribution to the magnetization gets redistributed across strings. In particular, increasing $|\Delta|$ favors large strings. In contrast, the prethermal evolution separately conserves the population of each string, and it shows a higher concentration of excitations in the small string sizes shown in the plot. Finally, we present in Supplementary Fig. 3 the evolution along the prethermal strokes of the distance from the prethermal state to a thermal state with the same energy and magnetization. The formula for the distance is the generalization of Supplementary Equation 9 to the presence of strings, $d(\rho_{\rm GGE}, \bar{\rho}_{\rm th}) = \left(\frac{\sum_j \int {\rm d}\lambda [\rho_{{\rm GGE};j}(\lambda) - \bar{\rho}_{{\rm th};j}(\lambda)]^2}{\sum_j \int {\rm d}\lambda [\bar{\rho}_{{\rm th};j}(\lambda)]^2}\right)^{1/2}$ where we make the sum over all the strings explicit for the sake of clarity. As for the Ising model, the relative distance at the end of the strokes is around the 40% for negative temperatures, and goes up to 60% for the first stroke at positive temperature, meaning the system is considerably far from equlibrium. Finally, we conclude by emphasizing that our study explores a wide range of finite cycles, consistently finding, beyond any numerical error, that prethermal cycles are more efficient at negative temperatures and, conversely, less efficient at positive temperatures.

SUPPLEMENTARY METHODS 1. NUMERICAL METHODS

This section summarizes the numerical methods we employed for the TBA and GHD equations. We keep the discussion compact, as these are standard methods, and refer to the literature for further details. A commented Mathematica notebook for the numerical solution of a cycle is provided on Zenodo [4].

Solving the TBA — The TBA and GHD equations are solved by discretizing the rapidity space on a grid. We consider $\{\lambda_i\}_{i=1}^N$ a discretization of $[-\pi/2, \pi/2]$ with $\lambda_i < \lambda_{i+1}$, with the convention that $\lambda_1 = -\pi/2$ and $\lambda_N = \pi/2$. We choose a flat discretization, but other choices are equally valid. Functions like energy, momentum, root density, and so forth are discretized with the midpoint rule $e_i(\lambda) \to e_{(i,j)} = e_i\left(\frac{\lambda_j+\lambda_{j+1}}{2}\right)$, where the index j runs over the rapidity discretization and i over the strings, which are truncated on a maximum cutoff $i \leq N_{\rm str}$. This truncation is standard, as strings are thermally activated and their length is exponentially suppressed. The scattering kernel is discretized by integrating it exactly on a finite interval

$$\varphi_{i,i'}(\lambda-\lambda') \to \varphi_{(i,j),(i',j')} \equiv \int_{\lambda_{j'}}^{\lambda_{j'+1}} \mathrm{d}\lambda'\, \varphi_{i,i'}\left(\frac{\lambda_j+\lambda_{j+1}}{2} - \lambda'\right). \qquad \text{(Supplementary Equation 25)}$$

The integral can be easily analytically computed from the definition. This discretization is convenient for integral equations in which the scattering kernel is convoluted with a smooth function, like the filling function, even if φ has sharp variations. As a last step, the couple of indexes (i,j) is converted into a single index $\mathrm{n} = j + i(N-1)$: the integral TBA equations (see Methods) are then discretized as

$$\varepsilon(\lambda) = \sum \beta_i q_i(\lambda) + \int \frac{\mathrm{d}\lambda'}{2\pi}\varphi(\lambda-\lambda')\log(1+e^{-\varepsilon(\lambda')}) \quad \to \quad \varepsilon_{\mathrm{n}} = \sum_i \beta_i q_{\mathrm{n},i}(\lambda) + \sum_{\mathrm{n}'} \frac{1}{2\pi}\varphi_{\mathrm{n},\mathrm{n}'}\log(1+e^{-\varepsilon_{\mathrm{n}'}}). \qquad \text{(Supplementary Equation 26)}$$

The discretized non-linear equations are then solved by standard methods, like the Newton–Raphson method. A similar discretization is also used for the dressing equations (defined in Methods), which become linear matrix equations and are solved with standard linear algebra methods.

Solving the strokes — In integrable models, the flow equations describing infinitesimal strokes (2) are conveniently reformulated in the quasiparticle basis. In particular, the prethermal strokes are equivalent to GHD evolution [3], whereas for the thermalizing stroke, we repeatedly solve the TBA equations with energy evolving on the adiabatic flow $\partial_\chi\langle H\rangle = \langle\partial_\chi H\rangle$. Hereafter, we discuss the numerical solution of the GHD equation within the method of characteristics [3]. We discuss it at the level of the exact GHD equations, as it is more transparent, and discretization in the rapidity space straightforwardly follows from Section . To illustrate the method, consider Eq. (9), which can be rewritten in terms of the fillings [3] as a convective equation:

$$\partial_\chi \vartheta(\lambda,\chi) + F^{\rm eff}(\lambda,\chi)\partial_\lambda\vartheta(\lambda,\chi) = 0\,. \qquad \text{(Supplementary Equation 27)}$$

The solution can be then implicitly expressed as:

$$\vartheta(\chi',\lambda) = \vartheta(\chi,\lambda(\chi',\chi)) \qquad \text{where} \qquad \lambda(\chi',\chi) = \lambda - \int_\chi^{\chi'} \mathrm{d}\xi\, F^{\rm eff}(\lambda(\xi,\chi),\xi). \qquad \text{(Supplementary Equation 28)}$$

Here, $F^{\rm eff}(\lambda,\chi)$ depends non-trivially on the filling, making the solution implicit, but it paves the way to a systematic discretization. A second-order $\mathrm{d}\chi$ algorithm is achieved by discretizing the integral in Supplementary Equation 28 by the midpoint rule. To this end, one discretizes the χ−evolution on integers and half-integer steps, i.e. $n\mathrm{d}\chi$ and $(n+\frac{1}{2})\mathrm{d}\chi$. Let us call $\vartheta_n(\lambda) \equiv \vartheta(n\mathrm{d}\chi,\lambda)$ and $\vartheta'_n(\lambda) \equiv \vartheta((n+\frac{1}{2})\mathrm{d}\chi,\lambda)$ the discretized fillings. Then, one evolves $\vartheta_n \to \vartheta_{n+1}$ by approximating Supplementary Equation 28 as $\lambda((n+1)\mathrm{d}\chi, n\mathrm{d}\chi) = \lambda - \mathrm{d}\chi F^{\rm eff}(\lambda,(n+\frac{1}{2}\mathrm{d}\chi)\big|_{\vartheta'_n}$, where the effective force is computed with the shifted filling ϑ'_n. Then, in the next step, ϑ_{n+1} is used to evolve $\vartheta'_n \to \vartheta'_{n+1}$, and then the step is repeated. Notice that, for consistency, the precision up to $\mathcal{O}(\mathrm{d}\chi^2)$ is maintained if interpolations in the rapidity space are at least of second order as well. While the first filling $\vartheta_{n=0}$ is determined by the initial conditions, the input for the first shifted filling $\vartheta'_{n=0}$ must be approximated: this can be done by considering the evolution for χ from 0 to $\mathrm{d}\chi/2$ as an evolution on its own, discretized with a small step $\mathrm{d}\chi' \ll \mathrm{d}\chi$ and using first-order discretizations for the first auxiliary step; see Supplementary Ref. [3] for details.

[1] G. B. Mbeng, A. Russomanno, and G. E. Santoro, The quantum Ising chain for beginners, SciPost Phys. Lect. Notes , 82 (2024).

[2] M. Takahashi, *Thermodynamics of one-dimensional solvable models* (Cambridge University Press, 2005).

[3] A. Bastianello, V. Alba, and J.-S. Caux, Generalized hydrodynamics with space-time inhomogeneous interactions, Phys. Rev. Lett. **123**, 130602 (2019).

[4] A. Brollo, A. del Campo, and A. Bastianello, Universal efficiency boost in prethermal quantum heat engines, 10.5281/zenodo.15125829 (2025).
